# Supplementary material for: Exposure to polystyrene nanoparticles induce disruption of mitochondrial homeostasis and impairs trophoblast cell invasion and migration via MDM2/ROCK1 pathway
Source: PLoS One. 2025 Dec 5;20(12):e0337568. doi: 10.1371/journal.pone.0337568 (PMC12680179; doi:10.1371/journal.pone.0337568)

Blot raw data

Figure 4-A

GAPDH 37KDa

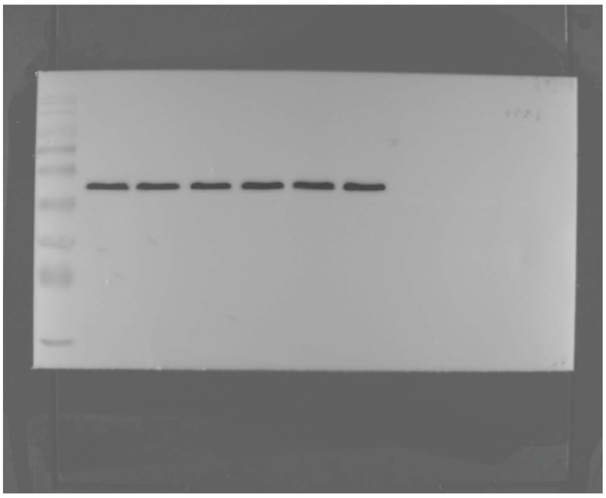

LC3I，II 14,16KDa

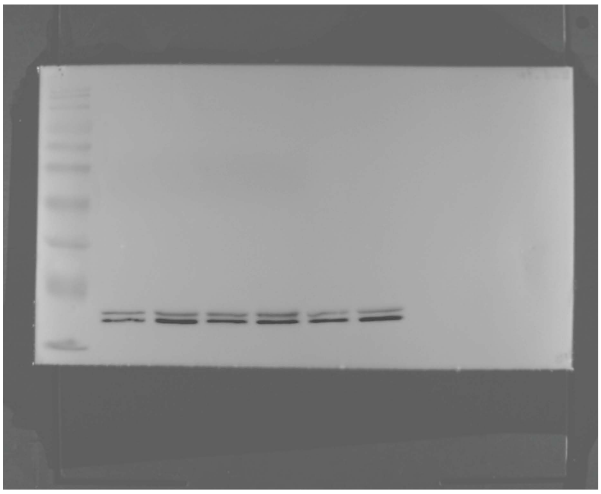

p62 62KDa

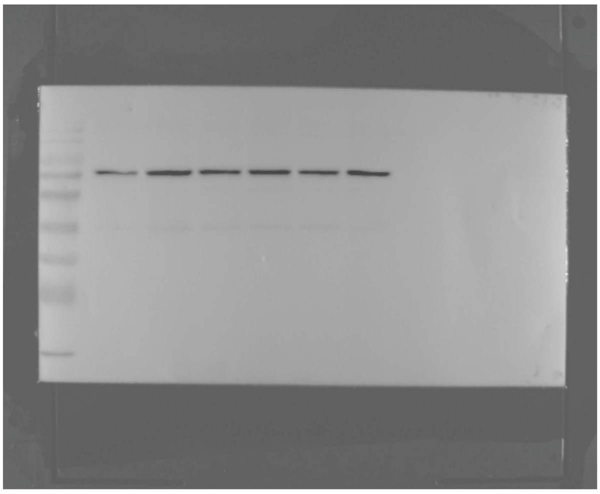

Pink1 45KDa

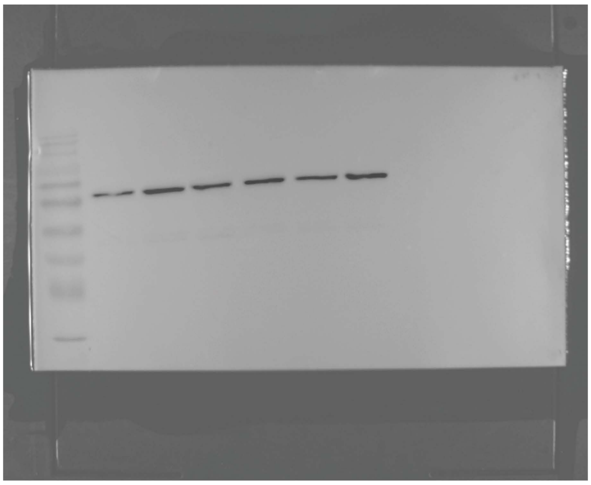

Figure 5-C

GAPDH 37KDa

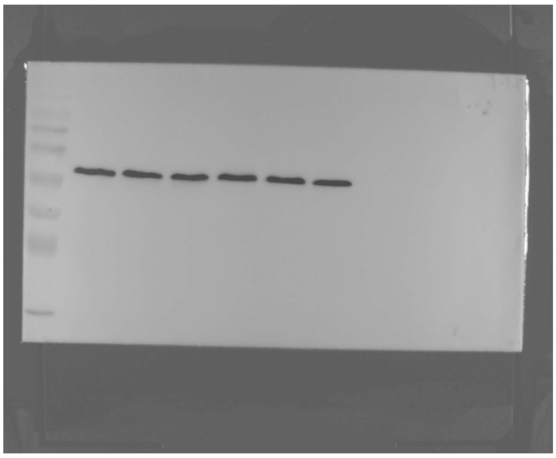

NDST1 70KDa

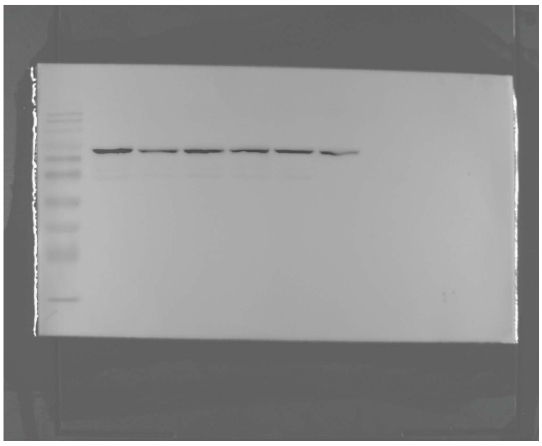

TSPAN4 26KDa

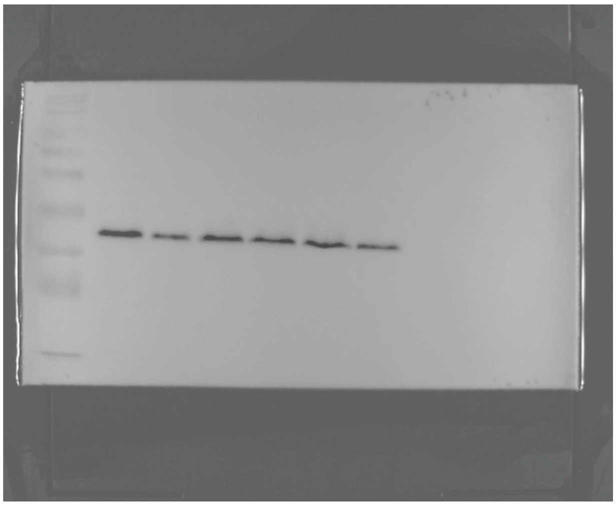

Figure 6-A

GAPDH 37KDa

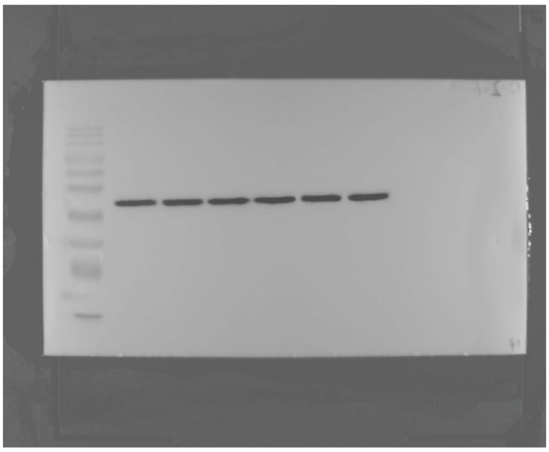

MDM2 90KDa

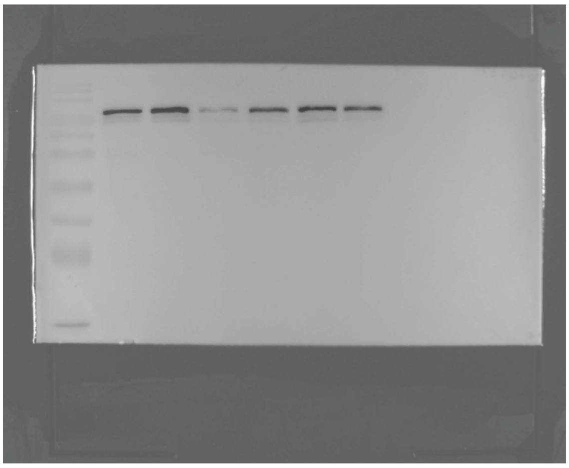

ROCK1 160KDa

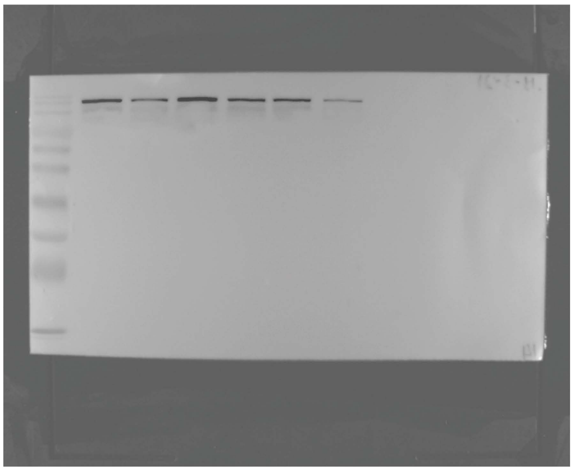

Figure 6-B

GAPDH 37KDa

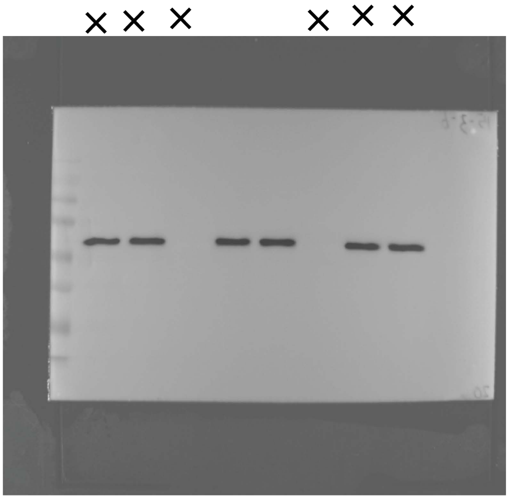

Input：MDM2 90KDa

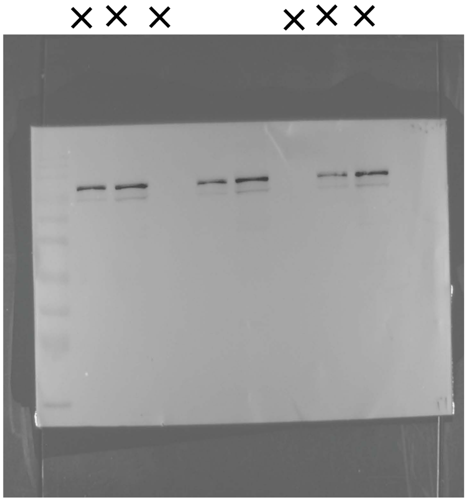

Input：ROCK1 160KDa

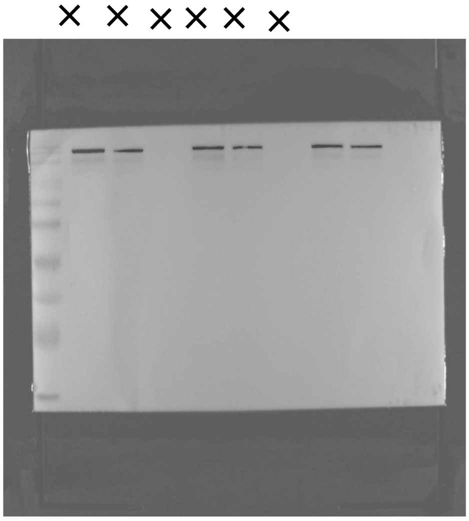

ip MDM2 90KDa

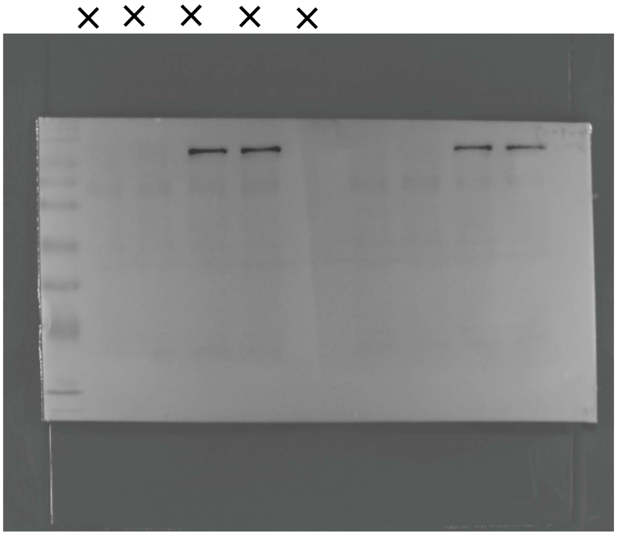

ip ROCK1 160KDa

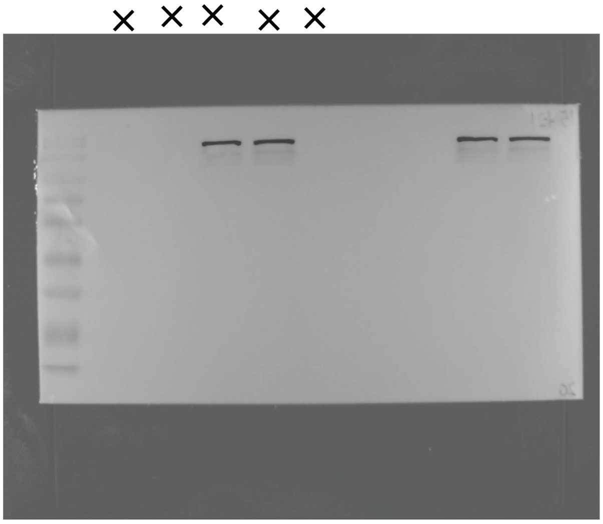

Figure 6-C

IB：ROCK1 160KDa

× × × × ×

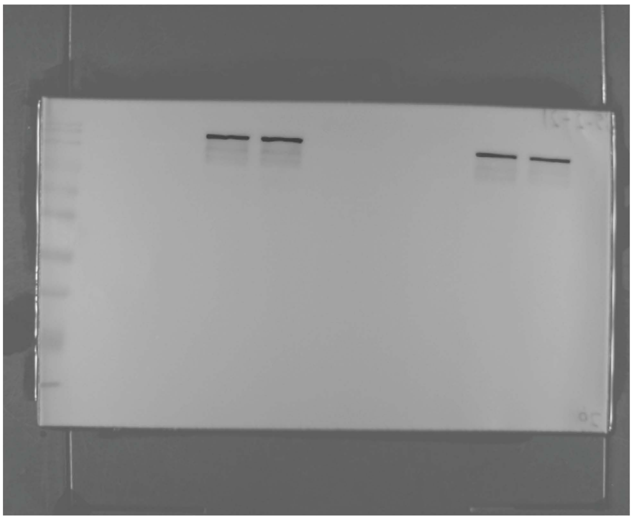

IB：UB

× × × × ×

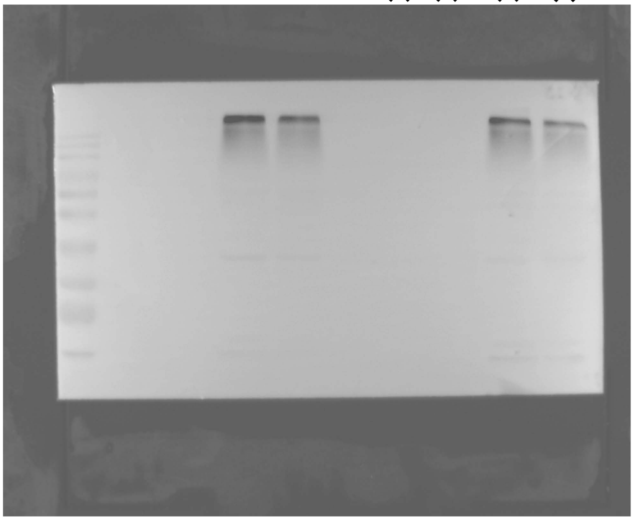

Supplement: S1 File — (PDF) [file pone.0337568.s001.pdf]
