# Supplementary figures and images for: Exposure to polystyrene nanoparticles induce disruption of mitochondrial homeostasis and impairs trophoblast cell invasion and migration via MDM2/ROCK1 pathway
Source: PLoS One. 2025 Dec 5;20(12):e0337568. doi: 10.1371/journal.pone.0337568 (PMC12680179; doi:10.1371/journal.pone.0337568)

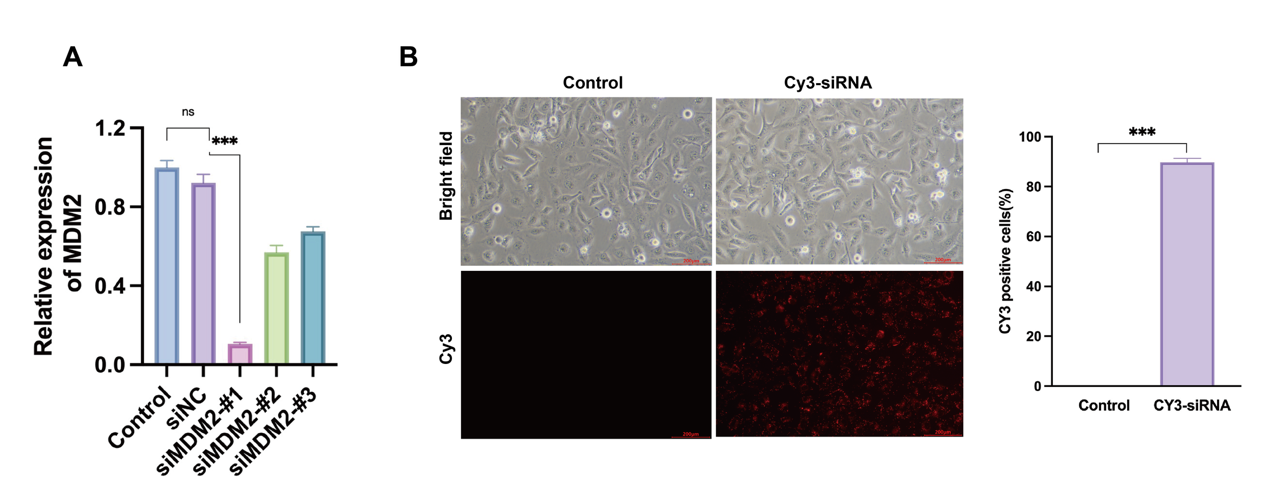

Supplement: S1 Fig — (A) RT-qPCR analysis of the knockdown efficiency of MDM2 using three different siRNAs. (B) Fluorescence microscopy images of Swan71 cells transfected with FAM-labeled siRNA. Scale bar = 200 μm. ***p < 0.001. (TIF) [file pone.0337568.s002.tif]
